# Supplementary material for: Impact of N-terminal dimerization on formin homology 1 domain polymer dynamics and actin assembly
Source: Biophys J. 2026 May 11;125(13):3299–316. doi: 10.1016/j.bpj.2026.05.006 (PMC13507409; doi:10.1016/j.bpj.2026.05.006)
Supplement: Supplementary file 1 [file mmc1.pdf]

Biophysical Journal, Volume *125*

Supplemental information

Impact of N-terminal dimerization on formin homology 1 domain polymer dynamics and actin assembly

Katharine Bogue, Bryan Christian, Margot E. Quinlan, and Jun Allard

Supplemental Material

Impact of N-terminal dimerization on formin homology 1 domain polymer dynamics and actin assembly

Katharine Bogue[1], Bryan Christian[1], Margot Quinlan[1,2,*], and Jun Allard[3,*]

[1]Department of Chemistry & Biochemistry, University of California Los Angeles, Los Angeles, CA 90095-1569 USA
[3]Department of Physics & Astronomy, Department of Mathematics, University of California Irvine, Irvine, CA 92697 USA
[2]Molecular Biology Institute, University of California Los Angeles, Los Angeles, CA 90095 USA
[*]Co-Corresponding authors

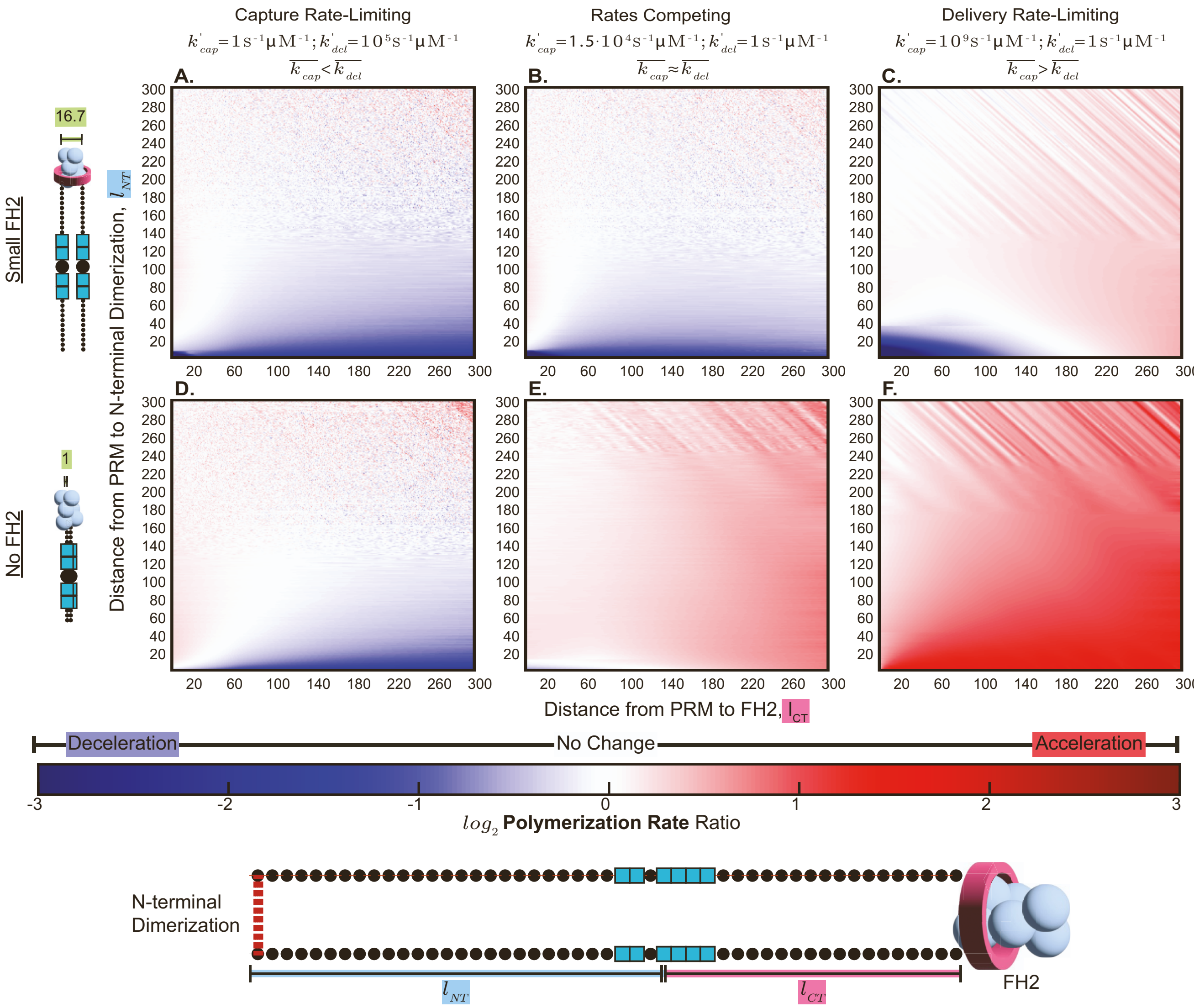

FIGURE S1 **N-terminal dimerization can result in acceleration or deceleration of polymerization based on parameter regime.** Combining the polymer behavior model (Fig. 2 and Fig. 3) with the kinetic model (Fig. 5), we can predict the impact of N-terminal dimerization on overall polymerization rates. The ratio (dimerized/non-dimerized) of polymerization rates for formins with a single binding site (PRM, proline-rich motif) are plotted as heatmaps with respect to the location of the binding site: distance from binding site to FH2 domain (l_{CT}, x-axis) and distance from binding site to N-terminal dimerization site (l_{NT}, y-axis). Color-map values range from blue to red, indicating a deceleration or acceleration of polymerization due to N-terminal dimerization, respectively. All regimes used the following parameters: $r^i_{\text{cap}} = 3.35\ \text{s}^{-1}$; $G = 1$; $C_{PA} = 1\ \mu\text{M}$. k'_{cap} and k'_{del} values were selected to showcase the behavior when capture is rate-limiting (a, d; $k'_{\text{cap}} = 1\ \text{s}^{-1}\ \mu\text{M}^{-1}$, $k'_{\text{del}} = 10^5\ \text{s}^{-1}\ \mu\text{M}^{-1}$), when delivery is rate-limiting (c, f;, $k'_{\text{cap}} = 1.5 * 10^4\ \text{s}^{-1}\ \mu\text{M}^{-1}$, $k'_{\text{del}} = 1\ \text{s}^{-1}\ \mu\text{M}^{-1}$), and when capture and delivery are competing (b, e;, $k'_{\text{cap}} = 10^9\ \text{s}^{-1}\ \mu\text{M}^{-1}$, $k'_{\text{del}} = 1\ \text{s}^{-1}\ \mu\text{M}^{-1}$). Simulations were run for 2 different FH2 domain sizes: (a-c) 16.7 amino acids and (d-f) 1 amino acid.

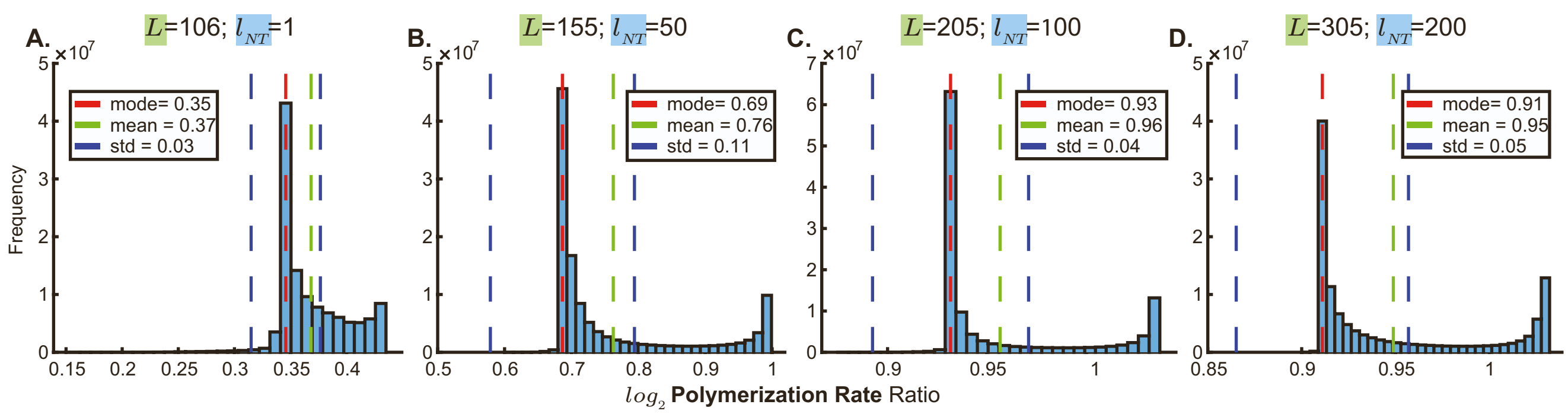

FIGURE S2 **Predicting the effect of N-terminal dimerization on Bni1 using MCMC posteriors.** Predicted polymerization rate ratios (dimerized/non-dimerized) for Bni1 using parameters from the posterior distribution of the MCMC fit to single binding site (PRM, proline-rich motif) Bni1 data. Simulations were run for 5 different binding site locations (l_{NT}) and FH1 domain lengths (L): (a) L = 106, l_{NT} = 1, (b) L = 155, l_{NT} = 50, (c) L = 205, l_{NT} = 100, (d) L = 305, l_{NT} = 200, The histogram mode (red dashed line), mean (green dashed line), and 1 standard deviation from the mode (blue dashed lines) are plotted on the histograms.

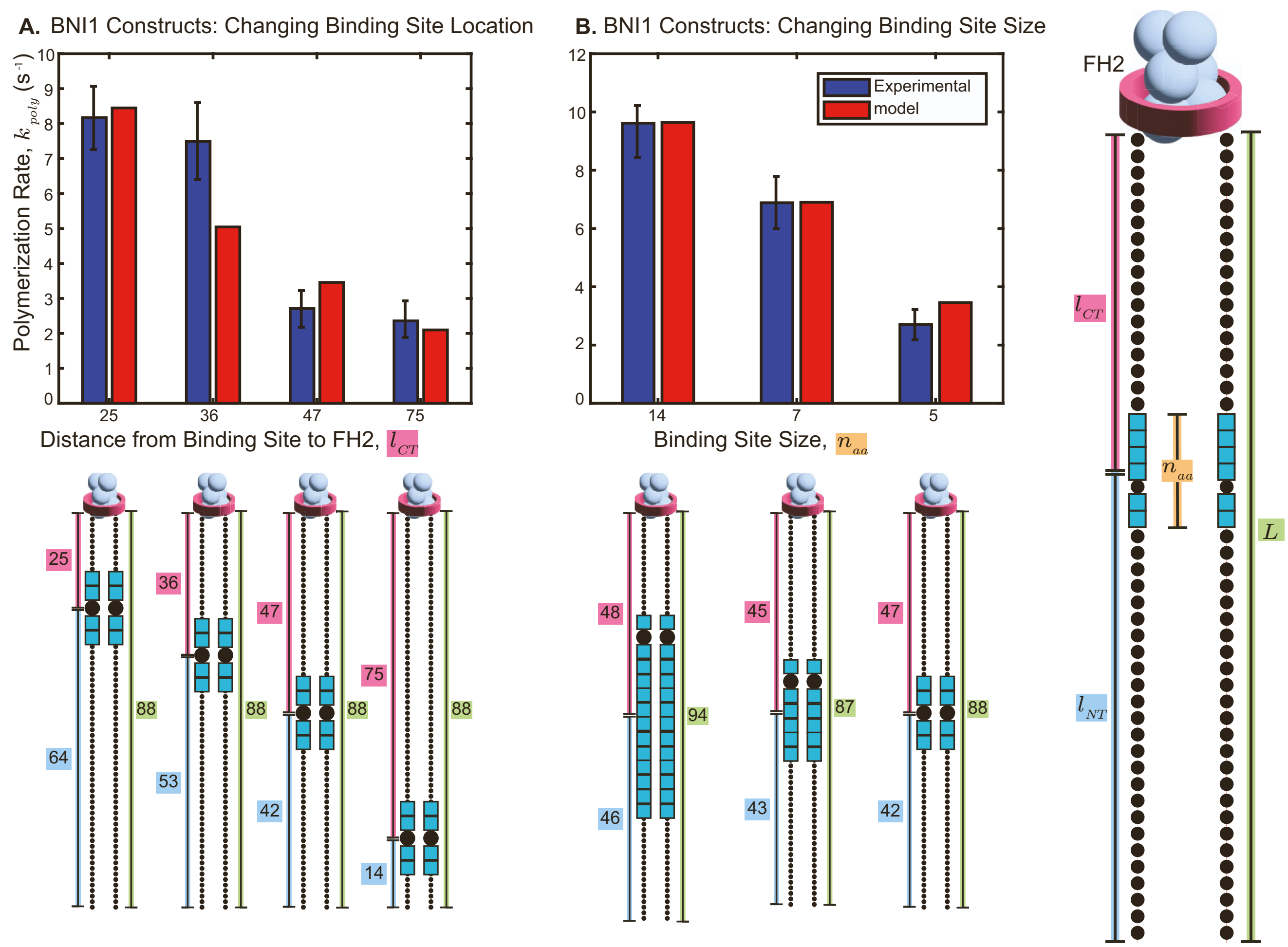

FIGURE S3 **Fit to experimental single binding site Bni1 data using best-fit parameters from MCMC fit.** Experimental (blue) and simulated (red) polymerization rates for single binding site (PRM, proline-rich motif) Bni1 constructs shown as side-by-side bar graphs. Constructs include those with the same binding site (PPAPP) at different locations along the FH1 domain (a) and those with different binding sites all at (roughly) the same location along the FH1 domain (b). Experimental values and error bars are taken from figures 3 and 4 of Courtemanche and Pollard (21) at profilin concentrations of 5µ M. Simulations were run using the best-fit parameters from the posterior distribution of the MCMC (Fig. 7): $k'_{\text{cap}} = 1.90 \times 10^3$ s^{-1} µ M^{-1}; $C_{PA} = 0.88$ µ M; $k'_{\text{del}} = 1.36 \times 10^{-3}$ s^{-1} µ M^{-1}; $r'_{\text{cap}} = 1.41 \times 10^3$ s^{-1}; $\epsilon = 0.57 k_B T$; $G = 0.5$. The best-fit parameters result in a RMSE of 0.639 subunits/s (compared to the experimental average SEM of 0.761 subunits/s) for the data changing binding site location (a) and a RMSE of 0.250 subunits/s (compared to the experimental average SEM of 0.769 subunits/s) for the data changing binding site site (b). Note that none of these constructs are N-terminally dimerized.

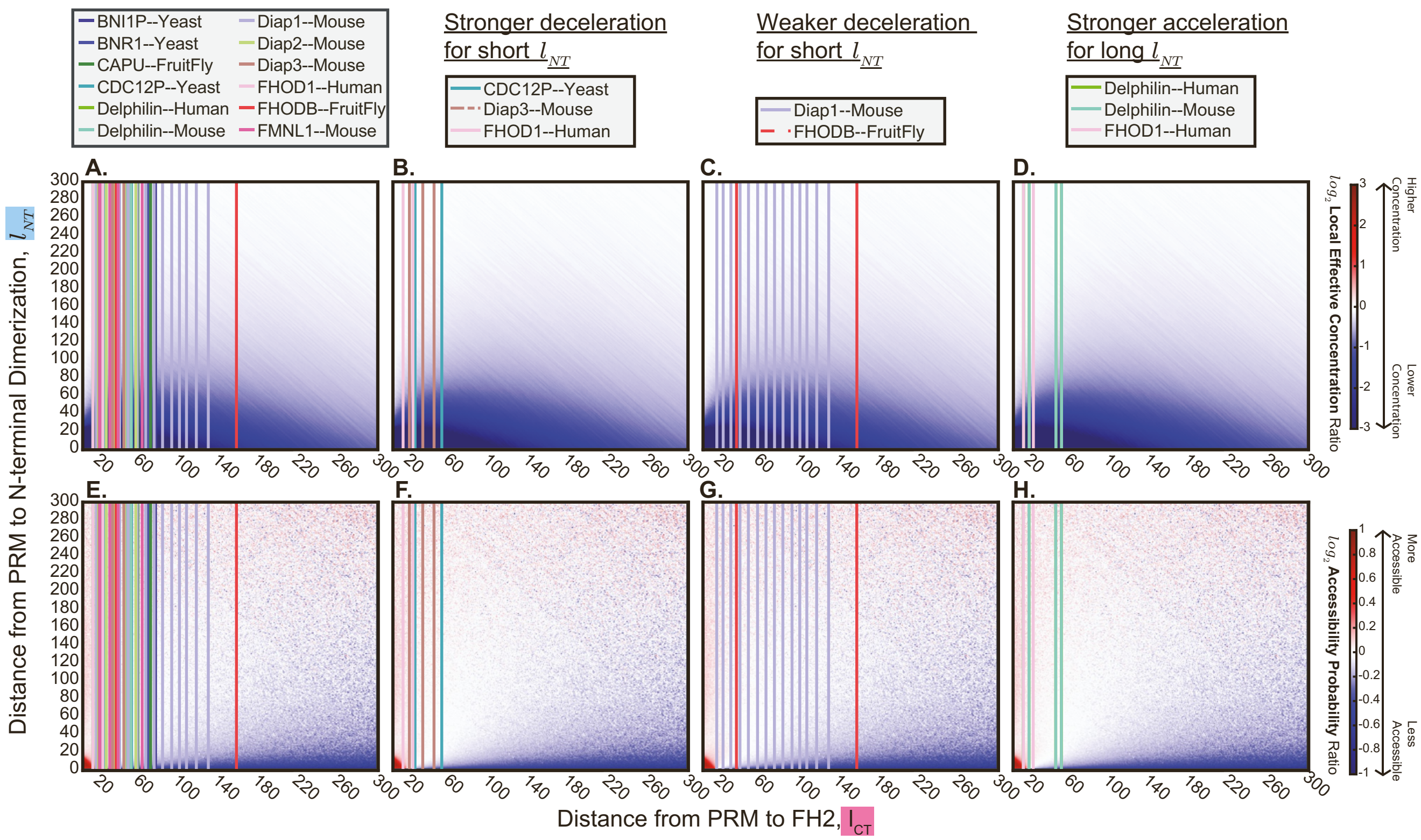

FIGURE S4 **Visualizing polymer behavior for binding sites based on the predicted effects of N-terminal dimerization.** The location of each binding site (PRM, proline-rich motif) for each formin (a,e) used in predictions (Fig. 8) is plotted over the heatmap of ratios (dimerized/non-dimerized) of polymer behavior parameters (Fig. 2c, Fig. 3c) with an FH2 domain size of 35.5 amino acids (from crystal structure of the Bni1 FH2 domain dimer, Xu et al. (22), PDB: 1UX5). Since the N-terminal dimerization domain locations for each formin are unknown, the binding site locations are plotted as vertical lines at the distance from the FH2 domain to the binding site (l_{CT}). (a-d) Simulated local effective concentration of binding sites at the growing end ($P_{\mathrm{r}}^{i,0}$). Higher (red) or lower (blue) local concentration of binding sites at the growing end enhances or impairs the rate of delivery, respectively. (e-h) Simulated binding site accessibility ($1 - P_{\mathrm{occ}}^{i}$). Increased (red) or decreased (blue) accessibility of binding sites enhances or impairs the rate of capture, respectively. Formins with extreme predicted effects of N-terminal dimerization are grouped by behavior and shown in additional plots: (b, f) formins that are predicted to experience greater deceleration when the N-terminal binding site is near the N-terminal dimerization domain (bottom left of Fig. 8b; Yeast CDC12P, Mouse Diap3, Human Fhod1); (c, g) formins that are predicted to experience less deceleration when the N-terminal binding site is near the N-terminal dimerization domain (top left of Fig. 8b; Mouse Diap1, Fruit Fly FhodB); (d, h) formins that are predicted to experience greater acceleration when the N-terminal binding site is far from the N-terminal dimerization domain (top right of Fig. 8b; Human Delphilin, Mouse Delphilin, Human Fhod1).

TABLE S1 Formins used in N-terminal dimerization predictions

Formin	UniProt ID	Gating Factor	Ref.	Number of binding sites (PRMs)	Mean binding site (PRM) Size	Number of Prolines	FH1 domain Length
Diap1–Mouse	O08808	1	(30)	14	6.57	92	181
Diap2–Mouse	O70566	0.2	(30)	5	8.8	44	82
Diap3–Mouse	Q9Z207	0.26	(46)	3	9	27	61
CAPU–FruitFly	Q24120	1	(30)	5	9.2	46	101
FMNL1–Mouse	Q9JL26	0.55	(47)	4	9.25	37	91
FHOD1–Human	Q9Y613	0.04	(48)	2	9	18	34
BNR1–Yeast	P40450	1	(49)	4	8.25	33	100
CDC12P–Yeast	Q10059	0.05	(30)	2	8.5	17	76
Bni1–Yeast	P41832	0.5	(30)	4	9.5	38	110
FHODB–FruitFly	Q9VSQ0	0.5	(50)	2	10.5	21	226
Delphilin–Human	A4D2P6	0.04	(51)	4	6.5	26	71
Delphilin–Mouse	Q0QWG9	0.03	(51)	4	6.5	26	71

TABLE S2 Polymer behavior of binding sites based on the predicted effects of N-terminal dimerization

Formins	FH1 Domain Sequence Feature	Predicted Binding Site Behavior	Predicted Polymerization Behavior
Yeast CDC12P, Mouse Diap3, Human Fhod1	Binding sites are close to the FH2 domain	Strong decreases in local effective concentration (bottom of Fig. S4b)	Strong deceleration when the dimerization domain is close to the N-terminal binding site (bottom left of Fig. 8b)
Mouse Diap1, FruitFly FhodB	N-terminal binding sites are very far from the FH2 domain	Weaker decreases in local effective concentration (bottom of Fig. S4c)	Weak deceleration when the dimerization domain is close to the N-terminal binding site (top left of Fig. 8b)
Human Delphilin, Mouse Delphilin, Human Fhod1	Binding sites are close to the FH2 domain	Consistent mild accelerations in accessibility (top of Fig. S4h)	Stronger acceleration when the dimerization domain is far from the N-terminal binding site (top right of Fig. 8b)

TABLE S3 Symbols

Rates		
Symbol	Description	Units
k_{poly}	polymerization rate for the full formin	s^{-1}
k^i_{poly}	polymerization rate for the binding site (PRM) located i amino acids away from the FH2 domain	s^{-1} $(\text{PRM})^{-1}$
k_{cap}	capture rate for the full formin	s^{-1}
k^i_{cap}	capture rate for the binding site (PRM) located i amino acids away from the FH2 domain	s^{-1} $(\text{PRM})^{-1}$
k_{del}	delivery rate for the full formin	s^{-1}
k^i_{del}	delivery rate for the binding site (PRM) located i amino acids away from the FH2 domain	s^{-1} $(\text{PRM})^{-1}$
r_{cap}	reverse capture rate for the full formin	s^{-1}
r^i_{cap}	reverse capture rate for the binding site (PRM) located i amino acids away from the FH2 domain	s^{-1} $(\text{PRM})^{-1}$
**Rate Constants**		
Symbol	Description	Units
k'_{cap}	capture rate constant	$s^{-1}\,\mu M^{-1}$
k'_{del}	delivery rate constant	$s^{-1}\,\mu M^{-1}$
r'_{cap}	reverse capture rate constant	s^{-1}
ϵ	reverse capture exponential scaling constant	n/a
**Rate-Modifying Variables**		
Symbol	Description	Units
P^i_{occ}	occlusion probability at the binding site (PRM) located i amino acids away from the FH2 domain	n/a
P^0_{occ}	occlusion probability at the FH2/barbed end	n/a
$1 - P^i_{occ}$	accessibility probability at the binding site (PRM) located i amino acids away from the FH2 domain	n/a
$1 - P^0_{occ}$	accessibility probability at the FH2/barbed end	n/a
$P^{a,b}_r$	probability density of location a at location b	μM
$P^{i,0}_r$	probability density of the binding site (PRM) located i amino acids away from the FH2 domain at the FH2 domain	μM
N	number of rods in a polymer simulation	n/a
C_{PA}	concentration of profilin-actin in solution	μM
G	gating factor (0-1)	n/a
**Length and Distance Notation**		
Symbol	Description	Units
n_{aa}	total number of amino acids in a binding site (PRM)	amino acids
n_p	number of prolines in a binding site (PRM)	amino acids
L	length (number of amino acids) of a formin from FH2 domain to NTDd	amino acids
l_{NT}	length (number of amino acids) from a binding site (PRM) to NTDd	amino acids
l_{CT}	length (number of amino acids) from a binding site (PRM) to FH2 domain	amino acids
L_k	Kuhn length	n/a

TABLE S4 Terms

Term	Description
polymerization	actin monomer addition to an actin filament (not including nucleation)
capture	binding site (PRM) binding to profilin-actin
delivery	binding site (PRM)-profilin-actin reaching the FH2 domain (loop closure)
polymer simulation	simulation of a random walk that produces polymer statistics; does not include formin-specific information
polymer statistics	the results of a polymer simulation
kinetic simulation	simulation of FH1-mediated polymerization, applying polymer statistics to a specific formin/construct
polymer model	a set of parameters, equations, set-up, etc for running polymer simulations
kinetic model	the set of steps (and thus equations) and their parameters used to calculate rates in a kinetic simulation
filament model	the method by which binding site (PRM) locations, binding site (PRM) sizes, FH1 domain N-termini, and FH1 domain C-termini are defined for a formin construct)
model	refers to the combination of kinetic, polymer, and filament models used to model FH1-mediated actin polymerization
NTD	N-terminal dimerization, the process
NTDd	N-terminal dimerization domain, the location
binding site (PRM)	proline-rich motif, the profilin-actin binding sites along the FH1 domain
occlusion probability	the probability that the simulated polymer conformation is such that the specified location is occluded (unavailable for binding)
accessibility probability	the probability that the simulated polymer conformation is such that the specified location is accessible (available for binding)
probability density	the probability that the simulated polymer conformation is such that one location is within a specified radius of another location (usually that a binding site is at FH2); proxy for local concentration
gating factor	the experimentally determined ratio of polymerization rates of FH2-only formin-mediated polymerization to actin alone; here, the probability that the FH2 domain is in a conformation that allows delivery
Kuhn length	the length of each rod in a freely-jointed chain
delivery location	the coordinates of the attachment point of the FH1 domain and the FH2 domain used when computing $P_r^{i,0}$ in polymer simulations
non-dimerized	refers to rates, parameters, simulations, etc where the FH1 domain is dimerized at the FH2 domain but not the NTDd
dimerized	refers to rates, parameters, simulations, etc where the FH1 domain is dimerized at both the FH2 domain and the NTDd
interruption	non-proline amino acid(s) within binding sites (PRMs)

VIDEO S1-2 Polymer dynamics simulations of non-dimerized FH1 domains. Video illustrations capturing 299 configurations of polymer dynamics simulations for non-dimerized FH1 domains with a length of 121 amino acids (the length of the Bni1 FH1 domain). The 299 configurations were generated by allowing the polymer simulations to run for 500000 iterations in order to equilibrate, and then recording the configurations of the polymer every 13 iterations for 3887 iterations. The simulations only include the FH1 domains (dark purple), but illustrative representations of the FH2 domain (pink torus) encircling the growing actin filament (light blue spheres) are included to orient the viewer. Terminal actin subunits are represented transparently in order to allow visualization of the FH1 domains. FH1 geometry is shown to scale, with each link having a length of 1 amino acid and the FH1 bases placed 35.5 amino acids apart.

VIDEO S1 Polymer dynamics simulations of non-dimerized FH1 domains (example 1).

VIDEO S2 Polymer dynamics simulations of non-dimerized FH1 domains (example 2).

VIDEO S3-4 Polymer dynamics simulations of dimerized FH1 domains. Video illustrations capturing 299 configurations of polymer dynamics simulations for dimerized FH1 domains with a length of 121 amino acids (the length of the Bni1 FH1 domain). The 299 configurations were generated by allowing the polymer simulations to run for 500000 iterations in order to equilibrate, and then recording the configurations of the polymer every 300 iterations for 89700 iterations. The simulations only include the FH1 domains (dark purple), but illustrative representations of the FH2 domain (pink torus) encircling the growing actin filament (light blue spheres) are included to orient the viewer. Terminal actin subunits are represented transparently in order to allow visualization of the FH1 domains. FH1 geometry is shown to scale, with each link having a length of 1 amino acid and the FH1 bases placed 35.5 amino acids apart.

VIDEO S3 Polymer dynamics simulations of dimerized FH1 domains (example 1).

VIDEO S4 Polymer dynamics simulations of dimerized FH1 domains (example 2).
